# Role of PI3K/Akt signaling in memory CD8 T cell differentiation

**DOI:** 10.3389/fimmu.2013.00020

**Published:** 2013-02-01

**Authors:** Eui Ho Kim, M. Suresh

**Affiliations:** Department of Pathobiological Sciences, University of Wisconsin-MadisonMadison, WI, USA

**Keywords:** memory T cell, PI3K/Akt, mTOR, FOXO, differentiation, metabolism

## Abstract

The clonal expansion, differentiation into effectors and establishing an immunological memory are crucial components of the adaptive immune response. Following the initial encounter with a pathogen, clonal CD8 T cell expansion yields at least two distinct populations of effector cells, short-lived effector cells (SLECs) and memory precursor effector cells (MPECs). SLECs are the terminally differentiated cells, which play an active role in pathogen clearance and undergo apoptosis once the pathogen is eliminated. In contrast, MPECs persist and give rise to self-renewing memory cells. These memory CD8 T cells maintain a state of heightened alertness and are poised to rapidly respond and swiftly clear the pathogen upon antigen re-encounter. As one of the goals of vaccination is to induce the development of these memory CD8 T cells, understanding the cellular and molecular basis of memory cell differentiation is critical to rational vaccine design. It is clear that memory differentiation is complex and involves multiple interrelated signaling pathways. It is influenced by factors such as the strength and duration of antigen receptor signaling and concurrent exposure to cytokines. Several signaling pathways that influence T cell fate have been recently described, and many culminate in the differential expression of specific transcription factors. Unfortunately, the mechanisms underlying the coordination and confluence of these signaling pathways remain largely unknown. In this review, we will discuss the role of the phosphatidylinositol 3-kinase signaling pathway as a central signaling node, and the function of Akt as a rheostat in orchestrating the differentiation of memory CD8 T cells.

## INTRODUCTION

CD8 T cells are highly specialized lymphocytes with a remarkable capacity to selectively target and kill tumor cells and cells infected with intracellular pathogens. As such, they play an important immunologic role in defending against tumors and infection by pathogenic organisms, particularly viruses and intracellular bacteria and protozoa. The development and maturation of antigen-specific CD8 T cells is a complex process involving numerous interrelated signaling pathways. This response has been most extensively characterized in animal models of acute viral infection, and this discussion is largely based on the findings from these models.

Antigen receptor engagement in the presence of appropriate co-stimulatory signals and exposure to cytokines such as type I interferons, activate CD8 T cells to undergo clonal expansion and differentiation into effector cells. At the peak of the T cell response, the expanded population of CD8 T cells is comprised of at least two distinct populations of effector cells, the short-lived effector cells (SLECs) and memory precursor effector cells (MPECs). SLECs are the terminally differentiated cytotoxic cells active in pathogen clearance and represent the majority of effector cells. CD8 T cell-mediated cytotoxicity depends upon recognition of specific viral antigens presented by class I major histocompatibility complex (MHC) molecules on the surfaces of infected cells. Antigen recognition induces the effector CD8 T cells to release molecules including perforin and granzymes, and cytokines such as interferon-gamma (IFNγ) and tumor necrosis factor-alpha (TNFα). Upon successful clearance of the pathogen, approximately 90% of effector cells including SLECs are eliminated by apoptosis.

The remaining 10% of effector cells represent the MPECs, which will differentiate into a self-renewing population of memory CD8 T cells. These memory CD8 T cells do not maintain a strong cytotoxic capacity, however, they persist for years in a state of heightened preparedness that enables them to rapidly proliferate and/or develop effector functions upon re-encounter of pathogens. This secondary response capacity of memory cells is significantly more rapid than the initial clonal expansion and leads to swift and expeditious control of the recurrent pathogen (Sallusto et al., 2010; Zhang and Bevan, 2011). Thus, the differentiation and maintenance of a functional memory CD8 T cell population provides effective, long lasting immunity.

The goal of vaccination is to prevent disease by pre-establishment of immunological memory similar to that induced by natural infection. Clearly, a detailed understanding of the cellular and molecular basis of memory cell differentiation is critical to rational vaccine design. Fortunately several important molecules involved in memory T cell differentiation have been identified and some have been well-characterized. However, the relationships between the individual molecules and the mechanisms by which their signaling is coordinated to ultimately make cell-fate decisions have been incompletely described or remain unknown. In this review, we will focus on the phosphatidylinositol 3-kinase (PI3K)/Akt signaling pathway and how it may integrate multiple extracellular cues and function as an immunologic rheostat that is able to spearhead a coordinated complex cellular response to govern this crucial differentiation of memory CD8 T cells.

## PI3K/Akt SIGNALING

Phosphatidylinositol 3-kinase/Akt signaling pathways exist in all mammalian cells and exert profound effects on multiple diverse processes including cell proliferation, survival, differentiation, migration, and metabolism. The importance of PI3K and its position as a central node in cell signaling pathways has been further demonstrated by studies which show that aberrant regulation of PI3K/Akt signaling is pathologic and results in diseases such as cancer and autoimmunity (Oak and Fruman, 2007; Jiang et al., 2009).

Phosphatidylinositol 3-kinase are divided into classes I, II, and III, based on structural and functional differences. Class I PI3Ks are further classified into class IA PI3Ks (PI3Kα, PI3Kβ, and PI3Kδ) and class IB PI3K (PI3Kγ), and they are well-characterized, while the significance and role of the other PI3K classes remains largely undetermined (Vanhaesebroeck et al., 2010). The class I PI3Ks are heterodimeric enzymes comprised of a regulatory subunit (p85) and a catalytic subunit (p110). Class IA PI3K’s place in the signaling chain is typically downstream of signals originating from receptor activation. Extracellular signals such as growth factors and cytokines bind to their receptors and stimulate receptor tyrosine kinases (RTKs). RTKs activate PI3K, which phosphorylates phosphatidylinositol-4,5-bisphosphate (PIP2) to generate phosphatidylinositol-3,4,5-trisphosphate (PIP3). PIP3 interacts with pleckstrin homology (PH) domain-containing target proteins such as Akt and phosphoinositide-dependent protein kinase (PDK1) on the inner leaflet of the plasma membrane.

**FIGURE 1:**
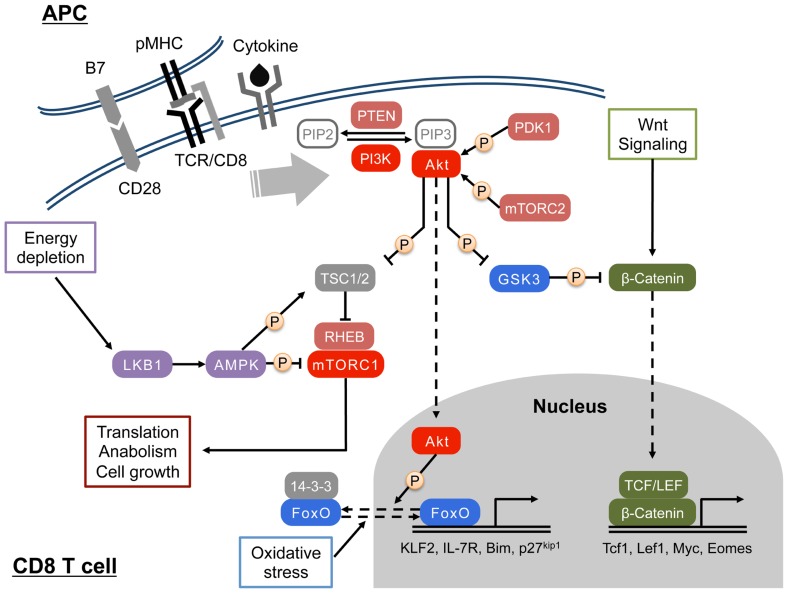
**Activation and function of PI3K/Akt signaling in CD8 T cell**. Upon engagement of the TCR by peptide/MHC I complex, PI3 kinase (PI3K) is activated in CD8 T cells. Signals from costimulation, cytokines, and chemokines can also activate the PI3K. At the plasma membrane, activated PI3K phosphorylates PIP2 to generate PIP3, which recruit PH domain-containing proteins such as Akt and PDK1. Full activation of the Ser/Thr kinase Akt requires phosphorylations by PDK1 and mTORC2. In the cytosol, Akt phosphorylates and inhibits TSC1/2, a negative regulatory complex of mTORC1, which in turn promotes mTORC1-mediated protein synthesis and cell growth through modulating S6K and 4E-BP. Cytosolic Akt also inhibits GSK3, and regulates glucose metabolism and the canonical Wnt/β-catenin pathway. In addition, Akt translocates to the nucleus, and triggers the nuclear exclusion of FOXO transcription factors that are important for cell quiescence and apoptosis. AMPK senses cellular energy status by interacting with ADP/AMP and LKB1 and regulates cellular metabolism by antagonizing mTORC1-mediated glycolysis.

Akt, also known as protein kinase B (PKB), has three isoforms – Akt1/PKBα, Akt2/PKBβ, and Akt3/PKBγ. Akt1 is ubiquitously expressed in various tissues including lymphocytes, whereas Akt2 is abundantly expressed and controls insulin-mediated glucose metabolism in muscle and adipocytes. Akt3 expression appears to be restricted to brain and testes (Hers et al., 2011). The kinase domains of all three isoforms have strong homology within kinase domains to the members of the protein kinase A, G and C families (AGC) kinase family (Manning and Cantley, 2007). At the plasma membrane, the interaction between PH domain of Akt and PIP3 results in important conformational changes in Akt, which enable subsequent modifications of Akt by PDK1. To achieve full activation, Akt has to be phosphorylated at T308 and S473 by PDK1 and mammalian target of rapamycin (mTOR) complex 2 (mTORC2), respectively (Alessi et al., 1997; Sarbassov et al., 2005; **Figure 1**).

Regulation of PI3K/Akt signaling interaction can occur via multiple mechanisms. Phosphatases such as phosphatase and tensin homolog (PTEN) and SH2 domain containing inositol 5′-phosphatase (SHIP) work as negative regulators of PI3K signaling by dephosphorylating PIP3 (Sly et al., 2003). Deletion of these molecules results in the elevated activation of PI3K signaling (Aman et al., 1998; Stambolic et al., 1998). Moreover, Akt activity is down-regulated by dephosphorylation at T308 and S473 by protein phosphatase 2 (PP2) and by the PH domain and leucine rich repeat protein phosphatases (PHLPP), respectively (Andjelkovic et al., 1996; Gao et al., 2005).

When fully activated, Akt becomes a powerful signaling molecule, which translocates from the cell membrane to the cytosol and nucleus where it can alter a large number of important signaling pathways. Akt modulation of these pathways is accomplished by serine and/or threonine phosphorylation of the targeted signaling molecules. Several examples common to most cells illustrate the potential impact of Akt activation. Akt phosphorylation of two negative regulators, tuberous sclerosis complex 2 (TSC2) and proline rich Akt substrate of 40 kDa (PRAS40), leads to mTORC1 activation. mTORC1 activation in turn controls protein synthesis, cell growth and metabolism (Laplante and Sabatini, 2012). Glycogen synthase kinase 3 (GSK3) is another direct substrate of Akt; by inhibitory phosphorylation of GSK3, Akt increases cellular glycogen synthesis. In addition, nucleic GSK3 regulates cell survival by phosphorylating cyclins and the transcription factors c-jun and c-myc (Hers et al., 2011). Apart from modulating the activities of mTORC1 and GSK3, Akt also phosphorylates and inactivates forkhead box O (FOXO) transcription factors in the nucleus thereby dampening the expression of FOXO target genes involved in proliferation, apoptosis, motility, and metabolism (Li et al., 2007; Hedrick et al., 2012; **Figure 1**). The PI3K/Akt pathway can indirectly control cellular functions by interfacing with other signaling pathways such as the canonical Wnt/β-catenin pathway, the nuclear factor κB (NF-κB) pathway, and the Janus kinase/signal transducers and activators of transcription (JAK/STAT) pathway (Okkenhaug and Vanhaesebroeck, 2003; Manning and Cantley, 2007; Delgoffe et al., 2011).

The preceding examples illustrate mechanisms by which PI3K/Akt signaling generally promotes cell growth and survival, while inhibition of PI3K/Akt signaling can growth and decrease cell survival. Although it is this association that has made the PI3K/Akt pathway an attractive target for anti-cancer therapies, and the proper regulation of many of these signaling pathways is also important for the generation of T cell responses. Because the PI3K/Akt signaling pathway is strategically positioned to influence so many aspects of the T cell response, the elucidation of its role in CD8 T cells is critical not only to understanding the immune response, but also to advancing rational vaccine design and development.

In T cells, the PI3Kδ and PI3Kγ isoforms are known to play critical roles during development. Deletion or inactivation of both isoforms during thymopoiesis results in a block at the CD4 CD8 double negative (DN) stage of T cell development. By contrast, PI3Kδ appears to be the isoform that is important for PI3K signaling in mature T cells (Okkenhaug and Vanhaesebroeck, 2003; Finlay, 2012). Although the biochemical mechanisms underlying the activation of PI3K in T cells are not fully elucidated (Chi, 2012), the PI3K/Akt signaling has important roles in the activation process as well as cytokine signaling in peripheral CD8 T cells (Kane and Weiss, 2003). In CD8 T cells, class IA PI3Ks are primarily activated by tyrosine kinase-associated receptors such as the T cell receptor (TCR), co-stimulatory and cytokine receptors. Signaling triggered by exposure to IL-12 and common gamma chain (γc) receptor-related cytokines such as IL-2, IL-7, IL-15, and IL-21 stimulates PI3K/Akt signaling pathway in CD8 T cells ( **Figure 1**). Among γc cytokines, IL-2 generates high and sustained levels of PIP3, whereas stimulation of PI3K by IL-15 is relatively weak and results in low levels of PIP3 (Cornish et al., 2006). In contrast, class IB PI3K is activated by G protein coupled receptors (GPCRs) such as chemokine receptors. Additionally, alterations in the cellular microenvironment that can regulate PI3K/Akt signaling pathway include the abundance of growth factors and immunomodulatory factors, and metabolic cues primarily derived from nutrients. Upon activation, T cells augment their metabolism to meet the high-energy needs of cellular processes such as proliferation, cytokine synthesis and secretion and cell-mediated cytotoxicity. While it is accepted that PI3K promotes the uptake of glucose and amino acids and enhance protein synthesis in activated T cells, the role of Akt in T cell metabolism during expansion has been questioned by a recent study (Macintyre et al., 2011). This study suggested that PI3K might regulate T cell metabolism by Akt-independent mechanisms, however, alterations in CD8 T cell trafficking and development of effector functions all require Akt activity. In contrast to Ag- and co-stimulation-mediated PI3K/Akt activation that strongly prime the initial proliferation and differentiation program of CD8 T cells, cytokines such as IL-2, IL-12, IL-15, and IL-21 might shape further differentiation after activation since each cytokine signaling is likely to work at distinct differentiation states (Schluns and Lefrancois, 2003; Cox et al., 2011).

## DIFFERENTIATION OF EFFECTOR AND MEMORY CD8 T CELLS

Understanding the differentiation of effector and memory CD8 T cells is an area of intense investigation in immunology. As discussed in the introduction, at the peak of clonal expansion there are two populations of effector T cells, the SLECs and MPECs. These two effector subsets can be identified by the differential expression of the cell surface molecules CD127 (the IL-7 receptor) and killer cell lectin-like receptor G1 (KLRG1) (senescence marker). The characteristic SLECs phenotype is CD127^LO^KLRG1^HI^ while the MPEC phenotype is CD127^HI^KLRG1^LO^. The SLECs are destined for deletion upon resolution of the infection and are highly prone to apoptosis. Because they also have diminished proliferative potential they are considered to be in a terminally differentiated state (Joshi et al., 2007; Sarkar et al., 2008). In contrast, MPECs persist beyond the resolution of the infection and ultimately differentiate into long-lived, self-renewing CD8 memory T cells. Tremendous progress has been made in deciphering the mechanisms underlying the disparate fates of these cells: apoptosis versus differentiation into memory CD8 T cells.

Differentiation of MPECs and SLECs is regulated by multiple mechanisms including asymmetric cell division, exposure to cytokines such as IL-2 and IL-12 and the strength/duration of TCR signaling (Joshi et al., 2007; Sarkar et al., 2007; Jameson and Masopust, 2009; Kalia et al., 2010). It has been reported that exposure of CD8 T cells to IL-2 or IL-12 promotes terminal differentiation into SLECs at the expense of MPECs (Joshi et al., 2007; Kalia et al., 2010). Additionally, it was reported that the duration and intensity of antigenic stimulation is a key factor that controls the magnitude of CD8 T cell response and the differentiation of memory CD8 T cells (Sarkar et al., 2007; Teixeiro et al., 2009; Zehn et al., 2009). In recent years, seminal studies from several laboratories have identified key transcription factors that regulate disparate fate of SLECs and MPECs. Notably, high levels of T-bet, Blimp-1, ID2, and XBP-1 promote differentiation of SLECs. By contrast, high levels of Eomes, Bcl-6, ID3, Mbd2, and Bmi-1 favor differentiation of MPECs (Rutishauser and Kaech, 2010). According to the current paradigm, the relative levels of the opposing transcription factors (e.g., T-bet and Eomes) and/or their mutually antagonistic activities (e.g., Blimp-1 and Bcl-6) might control the differentiation of SLECs and MPECs (Finlay and Cantrell, 2011; Zhang and Bevan, 2011). From the signaling point of view, apart from antigen receptor signaling, IL-12 produced by dendritic cells increases T-bet expression, which promotes terminal differentiation of effector CD8 T cells (Joshi et al., 2007). Moreover, sustained IL-2 signaling favors the differentiation of SLECs in association with elevated expression of T-bet and Blimp1 (Kalia et al., 2010). Eomes is required for sustaining CD8 T cell effector function, but promotes memory differentiation by antagonizing the effects of T-bet and increasing the expression of IL-15R (Intlekofer et al., 2005; Zhou et al., 2010).

These findings are consistent with the hypothesis that it is the collective signaling of the TCR, the IL-2 receptor, and the IL-12 receptor that alters expression levels of the cell-fate-determining transcription factors, which in turn govern the differentiation of memory CD8 T cells. It is important to note, however, that the complex circuitry underlying this fateful pathway remains poorly defined, even though its characterization appears to be fundamental to our understanding of CD8 T cell differentiation. It is clear that this circuitry must facilitate the integration of signals emanating from diverse receptors and signaling pathways. The TCR, IL-2 receptor and IL-12 receptor signaling have all been demonstrated to stimulate the PI3K/Akt signal transduction pathway. Therefore, PI3K/Akt is a logical target for investigation into the complex circuitry underlying CD8 T cell differentiation. Nevertheless, a strong case can be made that the cumulative strength of Akt activation in effector cells, controlled by signaling emanating from multiple receptors including TCR, IL-2 receptor and IL-12 receptors control the balance between terminal differentiation and generation of CD8 T cell memory.

## ROLE OF PI3K/Akt SIGNALING PATHWAY IN CD8 T CELL DIFFERENTIATION

Akt appears to be situated in a position to coordinate the convergence of the CD8 T cell-fate-determining pathways, and it has been clearly demonstrated to regulate diverse cellular processes impacting CD8 T cell fate. This has generated considerable interest in investigating its roles as well as those of its downstream effectors, mTOR, FOXOs, and GSK3 in CD8 T cell homeostasis (Araki et al., 2009; Kerdiles et al., 2009; Ouyang et al., 2009; Rao et al., 2010, 2012; Sullivan et al., 2012). Macintyre et al. (2011) examined the role of Akt in controlling the metabolism and development of effector functions of CD8 T cells *in vitro*. These studies provided important insights into how the strength and duration of Akt activation might regulate the trafficking and differentiation of effector CD8 T cells by controlling the cellular transcriptome. First, they demonstrated that high levels of Akt activation down-regulate the expression of adhesion molecules, CD62L, CCR7, and sphingosine-1-phosphate receptor (SIP), thereby redirecting the trafficking of effector CD8 T cells away from the secondary lymphoid tissues into the sites of inflammation. Conversely, low levels of Akt activation did not down-regulate the expression of these adhesion molecules and CD8 T cells continued to traffic into the lymph nodes, and express a transcriptome that resembles the one present in memory CD8 T cells. Second, it was demonstrated that proliferation can occur in the apparent absence of Akt, but Akt activation appears to be essential for development effector functions in activated CD8 T cells (Macintyre et al., 2011). Kim et al. (2012) also showed that terminal differentiation of CD8 T cells induced by sustained exposure to IL-2 was associated with higher Akt activation *in vivo*. They demonstrated that sustained Akt activation *in vivo* invoked a transcriptional program that favored terminal differentiation of CD8 T cells at the expense of CD8 T cell memory, consequent to excessive activation of mTOR, loss of FOXO activity and down-regulation of the Wnt/β-catenin pathway (Kim et al., 2012). It is unclear how constitutive Akt activation leads to down-regulation of Wnt pathway effectors Tcf1, Lef1, and Myc *in vivo*. Additionally, the effects of sustained Akt activation on the metabolic state of effector CD8 T cells warrant further investigation.

Exposure to cytokines such as IL-7 and IL-15 also stimulate the PI3K/Akt signaling pathway (Barata et al., 2004; Hand et al., 2010). Therefore, an interesting topic of discussion is the role of homeostatic cytokines such as IL-7 and IL-15 on the differentiation of CD8 T cells. One possible explanation is that the magnitude of PI3K/Akt signaling triggered by TCR signaling is much higher compared to stimulation with IL-7 and IL-15. Additionally, signaling triggered by IL-7 or IL-15 might activate the PI3K/Akt signaling, but the downstream activation of mTORC1 might be limited. Second, the phosphorylation sites on Akt will likely differ depending upon the nature of the stimuli, and therefore leads to drastically different outcomes. Third, the spectrum of signaling pathways triggered by antigen versus IL-7/IL-15 are likely to be different and the interplay between various pathways might dictate the cellular response. It is also worth noting that IL-7R is rapidly down-regulated by TCR ligation, and gets selectively re-expressed in memory precursors (Kaech et al., 2003). Although IL-15Rβ (CD122) expression is enhanced by activation, IL-15 signaling may not be strong early in the response because this subunit also functions as a co-receptor for IL-2 (Kalia et al., 2010). Further, exogenous administration of IL-7 or IL-15 fails to elicit dramatic effects pertaining to formation of memory CD8 T cells (Melchionda et al., 2005; Nanjappa et al., 2008). However, *in vitro* exposure of naïve or memory human CD8 T cells to IL-15 can induce proliferation and effector functions, in the absence of TCR signaling (Liu et al., 2002; Alves et al., 2003). It is worth emphasizing that these studies were performed *in vitro*, where naïve/memory T cells were exposed to presumably high and non-physiological concentrations of IL-15 (≥10 ng/ml). Although these studies clearly show that IL-15 at concentrations of ≥10 ng/ml can exert effects comparable to that of TCR signaling, it is unknown whether T cells are exposed to such concentrations *in vivo*, due to the limited availability of these cytokines. However, it should also be noted that specialized T cells do display immediate effector functions at mucosal sites such as the intestines, where IL-15 is available at higher concentrations (Fehniger and Caligiuri, 2001). Therefore, we hypothesize that signaling triggered by IL-15 in naïve or memory CD8 T cells can mimic the effects similar to those exerted by TCR signaling depending upon the concentrations of IL-15 in the immunological milieu. And we further propose that low levels of IL-7 or IL-15 may not exert pronounced effects on the differentiation program of CD8 T cells. Rather, they may promote the survival and proliferation of memory precursors.

## REGULATION OF CD8 T CELL MEMORY BY mTOR

One of the important downstream effectors for the PI3K/Akt signaling is mTOR, a serine–threonine kinase that has substantial sequence homology with the members of the PI3K family. Traditionally, mTOR is known as a nutrient sensor that regulates cell growth and protein synthesis, and is selectively inhibited by the immunosuppressive drug, rapamycin. Cellular mTOR is present as two distinct complexes: mTORC1 and mTORC2. The mTORC1 complex is composed of the proteins mTOR, Raptor, mLST8, PRAS40, and Deptor, and promotes protein translation through 4E-BP and S6K. The mTORC2 complex is composed of mTOR, Rictor, mLST8, and mSIN1, and mTORC2 is less sensitive to rapamycin than mTORC1 (Sarbassov et al., 2005). A heterodimeric complex consisting of TSC1 and TSC2 has been identified as a negative regulator of mTORC1 activity in T cells. TSC1/TSC2 complex maintains quiescence of naïve T cells by regulating cell size, cell cycle entry, and cell survival (Yang et al., 2011). Initiation of the PI3K/Akt signaling pathway inactivates TSC1/TSC2 and stimulates the small Ras-related GTPase Rheb, which in turn directly triggers mTORC1 activity (Laplante and Sabatini, 2009, 2012).

There is evidence that mTORC1 might limit the differentiation of memory CD8 T cells. Studies by Araki et al. (2009) showed that mTORC1 negatively regulates the differentiation of MPECs and their subsequent differentiation into memory CD8 T cells. Following an acute lymphocytic choriomeningitis virus (LCMV) infection, treatment with rapamycin during the expansion phase promoted MPEC formation and consequently, enhanced the number of memory CD8 T cells. Alternatively, when rapamycin treatment was restricted to the contraction phase, the phase of effector to memory transition was accelerated and the differentiation of central memory CD8 T cells was substantially increased. This effect appears to be CD8 T cell intrinsic since silencing Raptor expression in CD8 T cells largely recapitulated the effects of rapamycin treatment on memory formation. Rao et al. (2010) also reported that inhibition of mTORC1 activity by rapamycin *in vitro* enhanced the development of MPECs. Furthermore, terminal differentiation of effector cells induced by sustained Akt activation is at least in part due to hyper-activation of mTOR (Kim et al., 2012). In summary, mTORC1 activity promotes terminal differentiation of effector cells at the expense of memory precursors but the underlying mechanism remains to be determined. It is proposed that mTOR might promote terminal differentiation of effector cells by increasing the T-bet:Eomes ratio because, mTORC1 activation promotes the expression of the transcription factor T-bet and also suppresses the expression of Eomes (Rao et al., 2010; Li et al., 2011). How T-bet drives terminal differentiation of effector CD8 T cells and how mTOR modulates expression of T-bet and Eomes remain to be determined. As compared to mTORC1, relatively little is known about the role of mTORC2. mTORC2 regulates Akt activation by phosphorylation at S473 (Sarbassov et al., 2005) and enhances cell survival without activating mTORC1 (Chen et al., 2010). Whether mTORC2 has significant roles in orchestrating memory CD8 T cell differentiation awaits further investigation. Notably, mTOR is well known as an integrative metabolic sensor that is also regulated by 5′ AMP-activated protein kinase (AMPK; Powell and Delgoffe, 2010). The role of mTOR in T cell metabolism will be discussed later.

## REGULATION OF CD8 T CELL MEMORY BY FOXOs

Members of the FOXO family transcription factors are direct substrates of Akt. There are four FOXO members namely FOXO1, FOXO3, FOXO4, and FOXO6. While FOXO1, FOXO3, and FOXO4 are widely expressed, the expression of FOXO6 is restricted to the nervous system (Hedrick et al., 2012). Because FOXOs oppose cell cycle entry and promote apoptosis, they are considered as tumor suppressors (Paik et al., 2007). Additionally, FOXOs might promote organismal longevity by detoxifying reactive oxygen species and supporting DNA repair (Salih and Brunet, 2008). Peripheral T cells express FOXO1 and FOXO3, and it is becoming increasingly clear that these proteins play crucial roles in the maintenance of peripheral T cell homeostasis (Hedrick et al., 2012). In their active unphosphorylated form, FOXOs localize to the nucleus where they promote the expression of target genes that suppress cell cycle entry or promote apoptosis. Activated Akt phosphorylates FOXOs resulting in their nuclear exclusion and translocation to cytoplasm through interaction with the nuclear shuttle, 14-3-3 (Hedrick, 2009; Hedrick et al., 2012). However, exposure of cells to oxidative stress or nutrient deprivation can induce nuclear retention of FOXOs, thereby promoting the transcription of FOXO target genes. In addition to Akt, AMPK, c-jun N-terminal kinase (JNK), and MST1 are known to cause posttranslational modification of FOXOs (Ouyang and Li, 2011).

The role of FOXO1 and FOXO3 in regulating T cell homeostasis has been examined by ablating FOXO1 and/or FOXO3 in mice. In one study, global loss of FOXO3 led to lymphoproliferative disease and multi-organ inflammation, however, further studies have failed to reproduce these results (Lin et al., 2004; Dejean et al., 2009). Studies of LCMV infection in global and T cell-specific conditional FOXO3 null mice showed that FOXO3 might constrain T cell responses by both T cell-intrinsic and extrinsic mechanisms (Dejean et al., 2009; Sullivan et al., 2012). In studies by Dejean et al. (2009) increased accumulation of CD8 T cells in FOXO3 null mice during an acute LCMV infection was linked to overproduction of IL-6 from FOXO3-deficient dendritic cells. However, studies by Sullivan et al. (2012) suggested that FOXO3 might also limit the accumulation of LCMV-specific CD8 T cells by T cell-intrinsic mechanisms that include BIM-dependent apoptosis. By virtue of increased accumulation of CD8 T cells during the primary response, FOXO3 deficiency augmented the magnitude of CD8 T cell memory without affecting their phenotype or function (Sullivan et al., 2012).

While the functions of FOXO3 in T cells are largely consistent with its growth inhibitory properties in other cells, the role of FOXO1 in mature T cells is quite unique. FOXO1 controls multiple facets of T cells including trafficking, tolerance, and survival. First, unlike FOXO3, which promotes apoptosis of T cells (Sullivan et al., 2012), FOXO1 supports the survival of T cells by inducing the expression of the IL-7Rα chain, which promotes IL-7-induced Bcl-2 expression. Additionally, FOXO1 controls T cell trafficking by promoting the expression of the transcription factor KLF2, which in turn induces the transcription of molecules involved in trafficking, CD62L, CCR7, and S1P1 (Kerdiles et al., 2009; Ouyang et al., 2009). Unlike the seemingly opposing effects on T cell survival, FOXO1 and FOXO3 co-operatively protect against autoimmunity. Loss of FOXO1 and FOXO3 in T cells results in uncontrolled T cell activation and autoimmunity, which is at least in part linked to defects in the generation of regulatory T cells (Ouyang and Li, 2011). In addition, disruption of T cell homeostasis in the absence of FOXOs could result from dysregulated expression of p15^Ink4b^, p21^Cip1^, and p27^Kip1^ by itself and/or in association with TGF-β/Smad signaling pathway (Ouyang et al., 2010; Hedrick et al., 2012). More recently, *in vitro* studies of T cells by Rao et al. (2012) showed that FOXO1 might directly induce Eomes expression, indirectly repress T-bet expression, and promote memory CD8 T cell differentiation. Rao et al. (2012) also reported that *in vitro*-activated FOXO1-deficient CD8 T cells have diminished ability to survive after adoptive transfer into syngeneic mice. However, neither do we know how FOXO1 regulates T-bet expression nor it is clear how FOXO1 might support survival of memory CD8 T cells. It is worth investigating whether loss of IL-7R expression, consequent to loss of FOXO1 leads to demise of FOXO1-deficient memory CD8 T cells. Interestingly the cyclin-dependent kinase inhibitor p27^Kip1^, a major target gene for FOXOs curtails the primary expansion of CD8 T cells and limits the number of highly functional memory CD8 T cells during an acute LCMV infection (Singh et al., 2010). This phenotype has not been recapitulated either in FOXO3 or FOXO1 null mice (unpublished observations; Tejera and Suresh).

## CONTROL OF T CELL METABOLISM BY PI3K/Akt SIGNALING

During the phase of antigen-driven clonal expansion, CD8 T cells proliferate intensively with an estimated doubling time of 4–6 h (Murali-Krishna et al., 1998; Badovinac et al., 2007). In order to support such rapid proliferation and effector functions including cell-mediated cytotoxicity and cytokine production, activated CD8 T cells increase uptake of glucose, amino acids, and iron (Fox et al., 2005), and switch glucose metabolism from fatty acid oxidation (catabolism) to aerobic glycolysis and glutaminolysis (anabolism) by mechanisms orchestrated by transcription factor c-myc consequent to Akt/Erk1/2 activation (Wang et al., 2011). PDK1 but not Akt appears to be required for metabolic programming of activated CD8 T cells to aerobic glycolysis. While aerobic glycolysis may be required for clonal expansion and effector functions, effector CD8 T cells do switch back to catabolism during effector to memory transition (Prlic and Bevan, 2009). The metabolic switch to catabolism might be a necessary event for generation of CD8 T cell memory because defects in the fatty-acid oxidation pathway induced by TRAF6 deficiency can dramatically decrease memory T cell generation (Pearce et al., 2009). Interestingly, TRAF6-deficient CD8 T cells exhibit hyper-activation of PI3K/Akt signaling, which suggests a role for this signaling pathway in regulating fatty acid metabolism and generation of CD8 T cell memory (King et al., 2006). Pharmacological augmentation of AMPK activation (by metformin treatment) and suppression of mTORC1 (by rapamycin treatment) improve memory formation from TRAF6-deficient CD8 T cells (Pearce et al., 2009). This study confirmed another report, which showed that rapamycin treatment during contraction phase accelerated the differentiation of central memory cells, implicating PI3K/Akt/mTOR pathway in controlling CD8 T cell metabolism and differentiation of memory CD8 T cells (Araki et al., 2009). In a recent report, van der Windt et al. (2012) showed that IL-15 promotes the generation of memory CD8 T cells by supporting fatty acid oxidation and enhancing the mitochondrial respiratory capacity of CD8 T cells. While collective evidence support the idea that PI3K/Akt signaling pathway might regulate cellular metabolism and differentiation of memory CD8 T cells, further studies are clearly needed to fully decipher the underlying mechanisms.

## CROSS TALK BETWEEN PI3K/Akt AND OTHER SIGNALING PATHWAYS

### Wnt/β-CATENIN SIGNALING PATHWAY

Accumulating data supports the Wnt/β-catenin signaling pathway might be important for generation and maintenance of CD8 T cell memory. The expression of the Wnt target genes is dynamically regulated during a T cell response. Expression of *tcf7* (encodes Tcf1), *lef1*, and *myc* is highest in naïve and central memory CD8 T cells, but substantially down-regulated in SLECs (Kim et al., 2012; Xue and Zhao, 2012). Thus, terminal differentiation into SLECs is associated with the loss of Wnt target gene expression and high-level expression of these genes correlates with survival or quiescence (Driessens et al., 2011). Studies that involved constitutive expression of β-catenin or loss of function mutants indicated that clonal expansion of CD8 T cells might require down-regulation of Wnt/β-catenin signaling but survival and maintenance of memory CD8 T cells are Wnt/β-catenin-dependent, especially Tcf1 (Jeannet et al., 2010; Zhao et al., 2010; Zhou et al., 2010). Mechanistically, Tcf1 could support CD8 T cell memory formation by directly inducing the expression of transcription factor Eomes, which is critical for sustained expression of the IL-2 receptor β chain (CD122; Zhou et al., 2010). Whether continued action of Tcf1 is required for maintenance of memory CD8 T cells remains unknown. Studies from Restifo’s group suggested that augmented Wnt signaling consequent to GSK3β inhibition reduced terminal differentiation of effector cells and promoted development of memory CD8 T cells with stem cell-like properties (Gattinoni et al., 2009b). It is GSK3β that provides a conduit for crosstalk between Wnt signaling and the PI3K signaling pathway. GSK3β is one of the central regulators of canonical Wnt signaling pathway and it is a direct substrate for Akt. Akt phosphorylates and inactivates GSK3β resulting in stabilization and nuclear localization of cytosolic β-catenin. Surprisingly, however, instead of potent activation, constitutively active Akt resulted in the strong inhibition of the downstream effectors of the canonical Wnt signaling in effector CD8 T cells (Kim et al., 2012). The mechanisms underlying the suppression of Wnt/β-catenin signaling by constitutively active Akt in CD8 T cells are yet to be determined, but it was recently reported that FOXO1 binds to the intergenic region of Tcf1 gene and induces its expression in regulatory T cells, with the implication that the loss of FOXO activity might impair the expression of Tcf1 in CD8 T cells (Ouyang et al., 2012).

### NF-κB SIGNALING PATHWAY

The nuclear factor κB signaling pathway regulates immune cell survival and various facets of innate and adaptive immunity (Vallabhapurapu and Karin, 2009). In an un-stimulated state, NF-κB family of transcription factors remain in the cytosol as homo- or hetero-dimers in complexes with the inhibitor of κB (IκB) proteins. Upon exposure to ligands for toll-like receptors (TLRs) or cytokines such as TNF and type I IFNs, IκB is phosphorylated and degraded by IκB kinases (IKKs). Consequent to the degradation of IκB, NF-κB re-localizes to the nucleus and alters transcriptional activity (Vallabhapurapu and Karin, 2009). There is precedent for regulation of CD8 T cell memory by the NF-κB signaling pathway. Constrained NF-κB signaling not only diminished clonal expansion of CD8 T cells, but also resulted in defective CD8 T cell memory (Hettmann et al., 2003). Further, defective NF-κB signaling triggered by a mutant TCR lead to a substantive reduction in the formation of memory CD8 T cells (Teixeiro et al., 2009). It is unclear how NF-κB signaling regulates the generation of CD8 T cell memory. Although debatable, there is evidence that PI3K/Akt signaling might interact with the NF-κB pathway at multiple levels (Salminen and Kaarniranta, 2010). For example, Akt potentiates transactivation activity of NF-κB through IKKβ and p38 mitogen-activated protein kinase (MAPK). And, PDK1, a downstream kinase of PI3K, directly phosphorylates IKKβ and activates NF-κB signaling. More recently, it was reported that suppression of Akt during T cell activation reduced NF-κB binding to its target gene promoters and diminished the expression of TNF and IL-6 (Cheng et al., 2011). Therefore, it is possible that PI3K/Akt signaling further tunes the differentiation of CD8 T cells through NF-κB pathway.

### JAK/STAT SIGNALING PATHWAY

The Janus kinase/signal transducers and activators of transcription signaling pathway is the principal signaling mechanism conveying biochemical signals from many growth factors and cytokines (Schindler et al., 2007; O’Shea and Plenge, 2012). Stimulation of this pathway induces dimerization and translocation of STAT to the nucleus. In the nucleus, STAT functions as a trans-activator of numerous target genes involved in cell proliferation, differentiation, survival, and migration (Schindler et al., 2007; O’Shea and Plenge, 2012). There is evidence that cytokines such as IL-7 and IL-15 trigger the JAK/STAT signaling pathway and modulate the differentiation and homeostasis of memory CD8 T cells (Schluns and Lefrancois, 2003; Hand et al., 2010; Tripathi et al., 2010). There is scant direct evidence that PI3K/Akt signaling can influence JAK/STAT signaling, but a recent study has suggested that either mTORC1 or mTORC2 could differentially affect JAK/STAT signaling through regulating the expression of suppressor of cytokine signaling (SOCS) in mouse primary T cells (Delgoffe et al., 2011). Significant to memory CD8 T cell differentiation, loss of memory CD8 T cells induced by constitutively active Akt in CD8 T cells was associated with impaired STAT5 signaling in response to cytokines such as IL-2, IL-7, and IL-15 possibly due to hyperactive Akt-mediated inhibition of IL-7R and IL-2R β chain expression. Conversely, constitutively active STAT5 enhanced the generation and/or survival of memory CD8 T cells (Hand et al., 2010). Thus, it is possible that a balance between STAT and Akt signaling could determine the survival of memory CD8 T cells.

## THERAPEUTIC MODULATION OF THE PI3K/Akt PATHWAY TO ENHANCE CD8 T CELL MEMORY

It is becoming increasingly clear that vaccines against diseases caused by complex pathogens such as AIDS, tuberculosis, and malaria need to elicit potent humoral and cell-mediated immunity. CD8 T cell-dependent protective immunity depends upon the quantity, quality, and anatomical localization of memory CD8 T cells. Conventional approaches to enhance memory responses by vaccines include the use of different forms and/or doses of antigen, adjuvant, and boosting strategies (Sallusto et al., 2010). Despite decades of research, very few adjuvants are licensed for use in humans. In the US and Europe, only aluminum salts (alum), AS04 (aluminum hydroxide in combination with TLR 4 ligand monophosphoryl lipid A [MPL]), and oil-in-water emulsions (MF59, AS03, and AF03) have been approved for human use (Coffman et al., 2010; Nordly et al., 2011; Pulendran and Ahmed, 2011; Foged et al., 2012). But, none of these adjuvants are known to induce potent CD8 T cell memory. With an in-depth understanding of the signaling pathways that regulate CD8 T cell memory, it is conceivable that targeted immunotherapies could be developed to enhance the quantity and quality of CD8 T cell memory (Gattinoni et al., 2009a). Studies by the Ahmed and Pearce groups have already demonstrated the feasibility of utilizing pharmaceutical agents to augment CD8 T cell memory *in vivo* (Araki et al., 2009; Pearce et al., 2009). In studies by Araki et al. (2009) inhibition of mTORC1 activity by rapamycin treatment during expansion phase or contraction phase significantly improved the generation of memory CD8 T cells in terms of quantity and/or quality. Likewise, Pearce et al. (2009) showed that treatment of mice with rapamycin and AMP kinase activator metformin enhanced the differentiation of memory CD8 T cells by metabolically reprogramming effector CD8 T cells. Because the amplitude of Akt activation correlated with terminal differentiation of effector CD8 T cells, Kim et al. explored the possibility of Akt blockade as a therapeutic strategy to enhance CD8 T cell memory. Treatment of mice with the pan-Akt inhibitor A-443654 during the expansion phase reduced mTOR activation and significantly enhanced the number of memory CD8 T cells. There have been considerable efforts to develop selective Akt inhibitors as treatment options for cancer. It is challenging to develop selective Akt inhibitors because, not only does Akt kinase has three isoforms, but these isoforms are highly homologous to AGC kinases (e.g., PKA, PKC, and S6K). However, Merck & Co., Inc. introduced MK2206, an allosteric inhibitor of Akt. MK2206 possesses low nanomolar potency against all three Akt isoforms, and has recently entered a Phase I clinical trial in patients with solid tumors. It would be interesting to assess whether MK2206 can augment CD8 T cell memory to vaccinations. The use of pharmaceutics to enhance CD8 T cell memory may be more enticing for the field of adoptive tumor immunotherapy. For example, tumor-infiltrating lymphocytes can be reprogrammed by pharmaceutics during *in vitro* expansion prior to adoptive transfer into patients (Restifo et al., 2012). Since transfer of central memory T cells provided superior anti-tumor effect compared to effector memory or effector T cells, (Gattinoni et al., 2005; Klebanoff et al., 2005), pharmacological modulation to promote the differentiation of central memory CD8 T cells during *in vitro* expansion would greatly improve the efficacy of immunotherapy.

## CONCLUDING REMARKS

**FIGURE 2:**
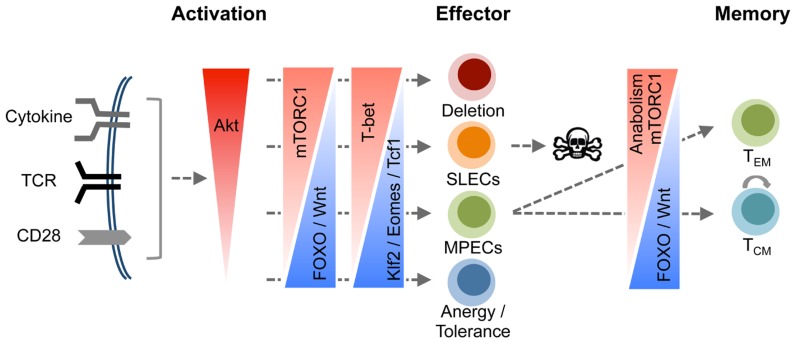
**A model for orchestration of CD8 T cell differentiation by the PI3K/Akt pathway**. Signals resulting from engagement of cell surface receptors including TCR, co-stimulatory molecules, and cytokine receptors converge to activate Akt, and the magnitude of Akt activation is a function of the cumulative signal strength from these receptors. Increase in the magnitude of Akt activation progressively drives cytotoxic T lymphocytes (CTLs) toward terminal differentiation. We propose a model where balanced Akt activation fosters development of effector functions without impeding the differentiation of MPECs and their descendent memory CD8 T cells. However, activation of Akt above a certain threshold drives differentiation of CD8 T cells into terminal effectors at the expense of MPECs by paralyzing a multitude of cell survival mechanisms including incapacitation of FOXO and the Wnt/β-catenin pathways, and stimulation of the mTOR pathway. Thus, Akt functions as a cellular fulcrum controlling distinct facets of the program that governs differentiation of antigen-activated CD8 T cells into terminal effector cells or memory CD8 T cells.

During an immune response, CD8 T cells are exposed to multiple extracellular signals, temporally and spatially, and the confluence of these signals not only determines the fate of antigen-activated CD8 T cells, they shape the quantity and quality of memory CD8 T cells. In this review, we have discussed how the PI3K signaling pathway might integrate multiple signals and control distinct facets of effector and memory differentiation by modulating specific downstream substrates of Akt ( **Figure 2**). The emerging consensus from published work is that strong Akt signaling is required for effective development of effector functions and guiding the effector cells away from the secondary lymphoid organs. By contrast, less intense Akt signaling might favor the differentiation of memory CD8 T cells. This forms the basis for the signal strength model for effector and memory differentiation ( **Figure 2**). However, this model leads to an unresolved question how and why only some activated CD8 T cells receive appropriate strength of signals and differentiate into memory cells? First, the duration and intensity of antigen receptor signaling depends on the: (1) nature and duration of infection; (2) expression of chemokine receptors (CXCR3 and CCR5) that regulate T cell/antigen-presenting cell (APC) interactions and the anatomical localization of the responding cells (Hu et al., 2011; Kohlmeier et al., 2011; Kurachi et al., 2011); (3) the stage of infection at which naïve T cells are recruited to the response (early responders versus latecomer cells; Badovinac et al., 2004; D’Souza and Hedrick, 2006). Second, the factors described above regulate the exposure of T cells to IL-2 and IL-12, which in turn promotes heterogeneity in the differentiation states. Third, diversification of effector CD8 T cells may be programmed at the first cell division, which occurs in an asymmetric manner. It is believed that the daughter cells that are in close proximity to the APC receives stronger TCR and co-stimulatory signals due to asymmetric receptor and cellular components and therefore differentiate into terminal effectors (Chang et al., 2007, 2011). Interestingly, the development of effector functions is closely associated with terminal differentiation, and it is currently unclear how these two processes are linked. Perhaps, transcription factors like T-bet promotes effector functions and at the same time controls genes that drive terminal differentiation. The molecular mechanisms underlying the T-bet-driven terminal differentiation of effector CD8 T cells including the identification of target genes for T-bet warrants further investigation. By the same token, while mTORC1 is known to drive terminal differentiation of effector CD8 T cells, the underlying mechanisms are still elusive. Furthermore, the role of FOXO1 in regulating CD8 T cell memory is unclear. Elucidating the specific roles of key players of the PI3K/Akt signaling pathway might lead to the development of pharmaceutics that can modulate diverse aspects of CD8 T cell memory.

## Conflict of Interest Statement

The authors declare that the research was conducted in the absence of any commercial or financial relationships that could be construed as a potential conflict of interest.
